# Notably small bronchial carcinoid accompanied by peripheral squamous cell carcinoma

**DOI:** 10.1002/rcr2.851

**Published:** 2021-09-23

**Authors:** Hiroyuki Miura, Jun Miura, Shinichi Goto, Keisei Tachibana, Tomoko Yamamoto

**Affiliations:** ^1^ Department of Thoracic Surgery Akiru Municipal Medical Center Akiruno Japan; ^2^ Department of Surgery Kyorin University School of Medicine Mitaka Japan; ^3^ Department of Respirology Akiru Municipal Medical Center Akiruno Japan; ^4^ Department of Pathology Tokyo Women's Medical University Akiruno Japan

**Keywords:** bronchial carcinoid, double cancer, lung cancer, thoracic surgery

## Abstract

Our report presents the smallest bronchial carcinoid thus far associated with peripheral squamous cell carcinoma. A 79‐year‐old Japanese man presented with an abnormal shadow on chest radiography. Chest computed tomography revealed an ill‐defined nodule at the left S9a area without lymph node involvement. At the time of bronchoscopy, a nodule of ~1 mm in diameter with a smooth surface was observed at the orifice of the left B8. Left lower lobectomy was performed. Pathologically, the S9a tumour was keratinizing squamous cell carcinoma with surgical stage IA3, and the left B8 tumour was a typical bronchial carcinoid. The patient was alive without recurrence 2 years after the surgery. Cases of combined carcinoid and lung cancer are rare. It may be possible to identify bronchial carcinoids early by careful observation of the bronchi during bronchoscopy for peripheral neoplastic lesions.

## Graphical abstract

Our report presents the smallest bronchial carcinoid thus far associated with peripheral squamous cell carcinoma. Cases of combined carcinoid and lung cancer are rare. It may be possible to identify bronchial carcinoids early by careful observation of the bronchi during bronchoscopy for peripheral neoplastic lesions.

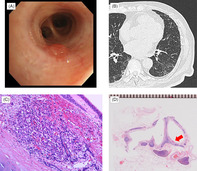

## INTRODUCTION

Enlarged bronchial carcinoids cause atelectasis, and patients may require bronchoplasty during surgery. Bronchial carcinoids are rarely diagnosed early, and there are a few reports on bronchial carcinoids in association with lung cancer. To the best of our knowledge, our case involves the smallest bronchial carcinoid thus far in association with peripheral squamous cell carcinoma.

## CASE REPORT

**FIGURE 1:**
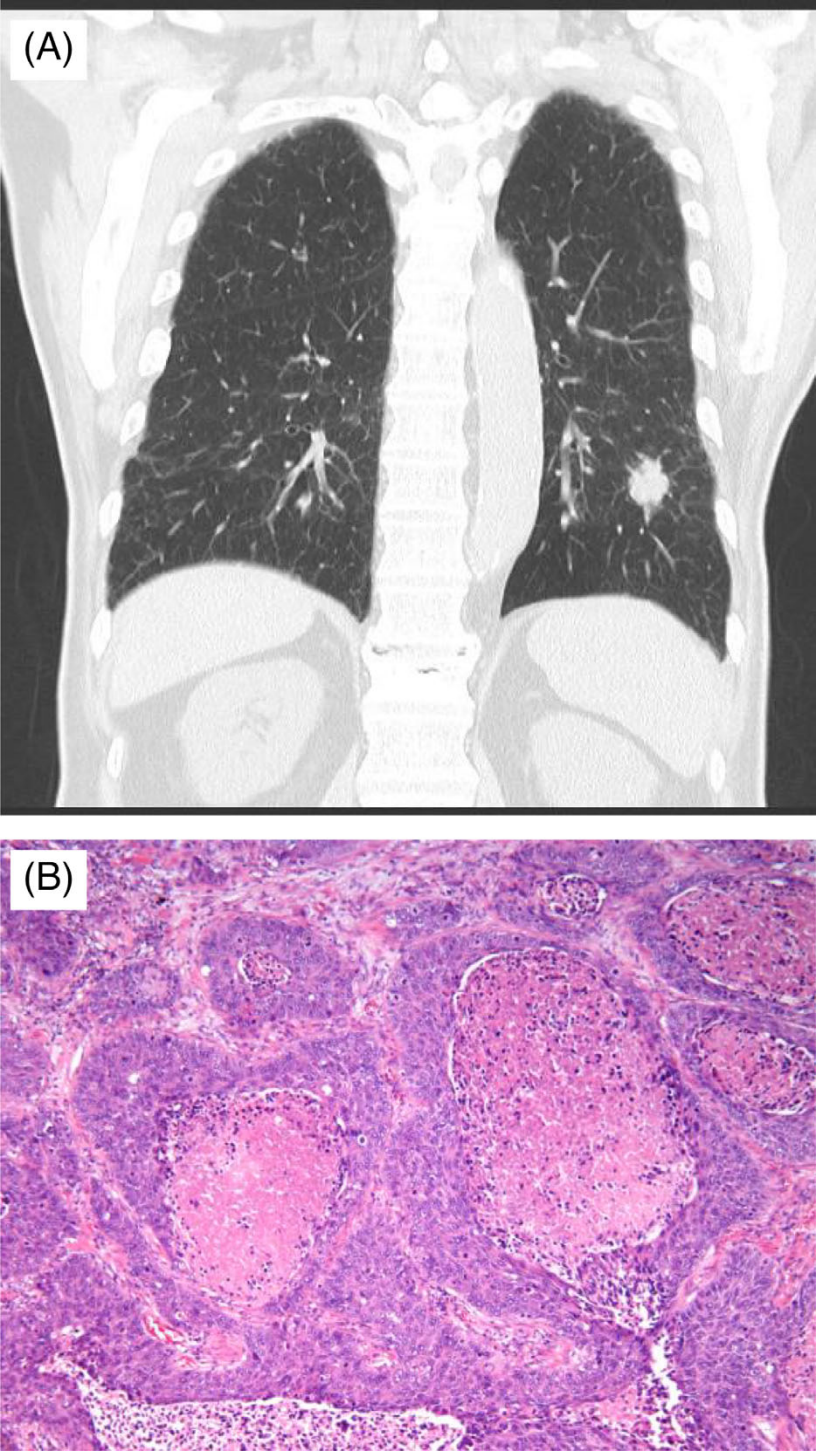
(A) Chest computed tomography showed an ill‐defined nodule of ~25 mm in diameter at the left S9a area without lymph node involvement. (B) The pathological specimen revealed keratinizing squamous cell carcinoma (haematoxylin and eosin ×20)

A 79‐year‐old Japanese man presented with an abnormal shadow on chest radiography during an annual check‐up. His family history was unremarkable. The patient had been treated for diabetes, and he was a current smoker (smoking index of 3680). The chest radiograph showed an ill‐defined nodule of ~25 mm in diameter in the left lower lung field. Chest computed tomography revealed an ill‐defined nodule of ~25 mm in diameter in the left S9a area without lymph node involvement (Figure 1A).

**FIGURE 2:**
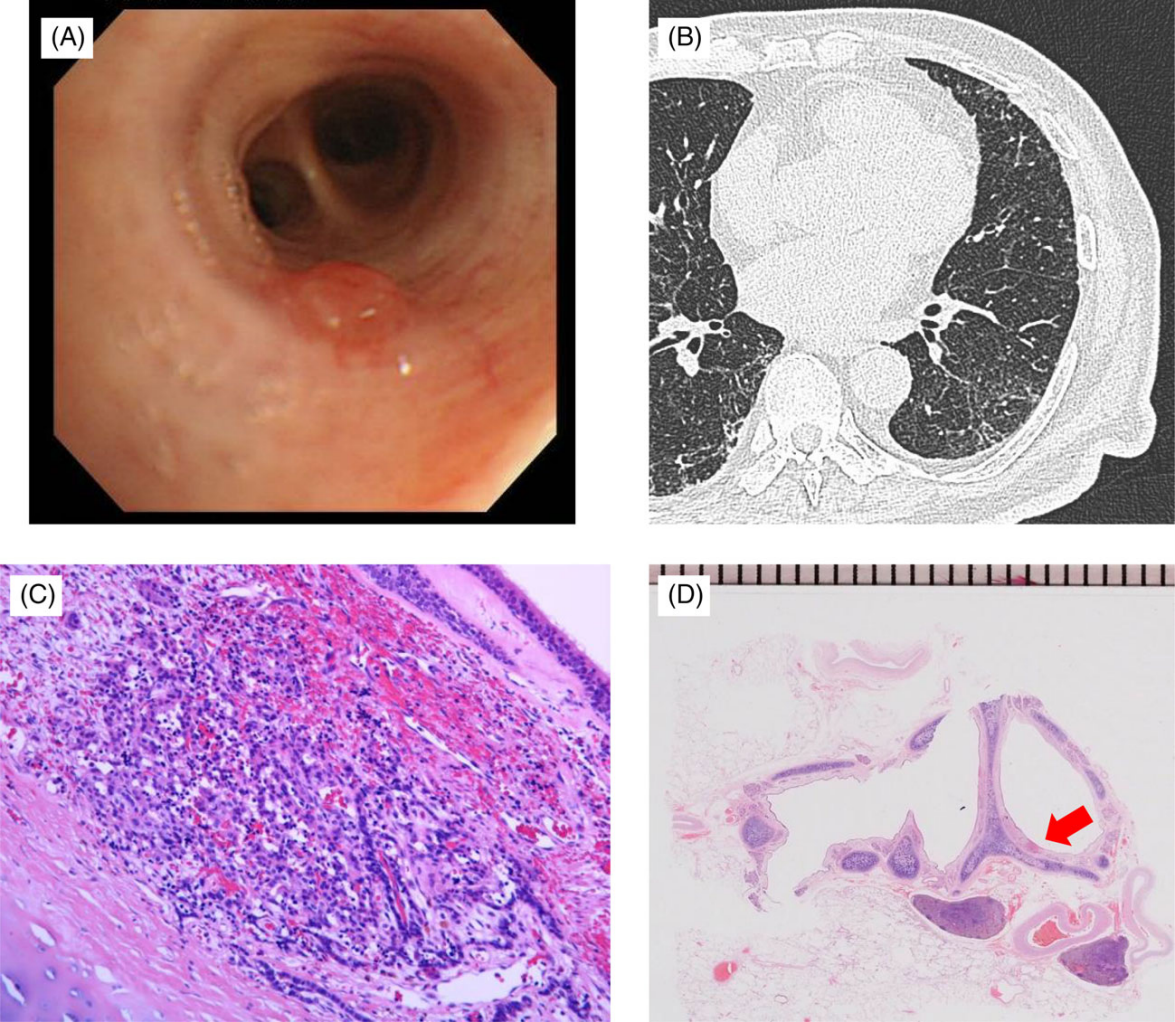
(A) Bronchoscopy showed a smooth surface nodule of ~1 mm in diameter at the orifice of the left B8a. (B) The bronchial tumour cannot be detected on the chest CT image. (C) A tumour of ~1 mm in size was observed at the entrance of the left B8 on the loupe image. (D) The B8 tumour showed a ribbon‐like arrangement suggesting a typical carcinoid tumour (haematoxylin and eosin ×20)

Blood examinations showed a slightly elevated carcinoembryonic antigen (5.9 ng/ml) but normal ranges of cytokeratin fragment and progastrin‐releasing peptide. The blood glucose level was high (164 mg/dl), with 9.1% HbA1c. Other than renal dysfunction with a creatinine level of 1.10 mg/dl and blood urea nitrogen level of 28.3 mg/dl, the haemogram and renal and hepatic functions were within normal ranges. Squamous cell carcinoma was detected via transbronchial lung biopsy. At the time of bronchoscopy, a nodule of ~1 mm in diameter with a smooth surface was observed at the orifice of the left B8a (Figure 2A). Pathological diagnosis of the tumour revealed typical carcinoid. The bronchial tumour cannot be detected on the chest CT image (Figure 2B).

After controlling hyperglycaemia, left lower lobectomy with mediastinal lymph node dissection was performed based on the diagnosis of cT1cN0M0 stage IA. Pathology revealed a 23 × 23 × 23 mm (same invasive dimension) tumour, which was a keratinizing squamous cell carcinoma (Figure 1B) without lymph node metastases or pleural involvement. The surgical stage was IA3 with pT1cN0M0. Moreover, a tumour (0.8 mm in diameter) was located at the orifice of the left B8 and was limited in the bronchial mucosa without invasion of the bronchial cartilage (Figure 2C). A ribbon‐like arrangement with positivity for chromogranin A and synaptophysin was observed. The Ki67 labelling index was <1%. Necrosis or mitosis was not observed. Finally, typical bronchial carcinoid was diagnosed (Figure 2D). The squamous cell carcinoma did not include any carcinoid component at all. The patient was alive without recurrence 2 years after the surgery.

## DISCUSSION

Carcinoid tumours are neuroendocrine tumours and account for <1% of all lung cancers. Carcinoid tumours are commonly located in the bronchus. Peripheral carcinoid tumours located more peripherally than subsegmental bronchus are described in 40% of cases. Carcinoids occur frequently in people who are aged <60 years, in women and in the white population. Typically, carcinoids are not related to tobacco smoking^1^; however, our patient was a non‐white older male who was a heavy smoker.

Cases of co‐occurring carcinoid and lung cancer are notably rare. To the best of our knowledge, only 17 cases except for the current case have been reported. In five cases, carcinoid tumour and non‐small‐cell carcinoma existed in the same tumours. Four carcinoid tumours formed collision tumours with two adenocarcinomas and two squamous cell carcinomas. One carcinoid tumour existed in the central scar of an adenocarcinoma.^2^ In six cases, the tumours consisted of peripheral carcinoid and non‐small‐cell carcinoma that were not in the same location as the carcinoid. The non‐small‐cell carcinomas included two adenocarcinomas, one squamous cell carcinoma, one adenosquamous carcinoma plus adenocarcinoma and one adenocarcinoma plus squamous cell carcinoma. In the remaining cases, squamous cell carcinoma was observed in the dissected lymph node with an unknown primary tumour.^3^ The remaining six cases were composed of bronchial carcinoid and peripheral adenocarcinoma; small‐cell carcinoma was also observed in one case.^4^ To the best of our knowledge, a combination of bronchial carcinoid and peripheral squamous cell carcinoma was only noted in the current case. In all bronchial carcinoid cases mentioned above, the bronchi were obstructed or narrowed by the tumours, and cases with secondary changes such as atelectasis in the periphery were also reported. Our case was the smallest bronchial carcinoid, being ~1 mm in maximum diameter.

Both tumours in our case were surgically resectable because they were in the same lobe and accompanying bronchus. If they had been in different bronchi and were unresectable, neodymium‐doped yttrium aluminum garnet (Nd‐YAG) laser would have been effective. Sutedja et al. reported on six bronchial carcinoids treated using Nd‐YAG laser. The resected specimens treated after the bronchoscopic therapy showed no residual carcinoids.^5^

The mechanism of bronchial carcinoid development is unclear, but it is believed to arise from Kulchitsky cells. In our case, we consider that some mechanism led to the carcinoid development alongside the squamous cell carcinoma due to heavy smoking. The carcinoid originated inside the smooth muscle layer under the bronchial mucosa. It may be possible to identify bronchial carcinoids early by careful observation of the bronchi during bronchoscopy for peripheral neoplastic lesions. Additional reports of small carcinoids, such as our case, are required for more detailed studies of carcinoids.

## CONFLICT OF INTEREST

None declared.

## AUTHOR CONTRIBUTION

Dr Hiroyuki Miura, Dr Shinichi Goto and Dr. Tomoko Yamamoto were involved in the conception and design of the work and the acquisition and analysis or interpretation of data. Dr Jun Miura and Dr Keisei Tachibana drafted the work and revised it critically for important intellectual content. All authors contributed to the final version of this manuscript and approved it to be published.

## ETHICS STATEMENT

The authors declare that appropriate written informed consent was obtained for publication of this case report and accompanying images.
